# Vitamin D Deficiency among Patients with Tuberculosis: a Cross-Sectional Study in Iranian-Azari Population

**Published:** 2019-01

**Authors:** Masoud Nouri-Vaskeh, Sepehr Sadeghifard, Parviz Saleh, Jafar Farhadi, Mahan Amraii, Khalil Ansarin

**Affiliations:** 1 Connective Tissue Diseases Research Center, Tabriz University of Medical Sciences, Tabriz, Iran; 2 Tuberculosis and Lung Diseases Research Center, Tabriz University of Medical Sciences, Tabriz, Iran; 3 Kidney Research Center, Tabriz University of Medical Sciences, Tabriz, Iran; 4 Molecular Medicine, Faculty of Advanced Medical Sciences, Tabriz University of Medical Sciences, Tabriz, Iran.

**Keywords:** Vitamin D Deficiency, 25-hydroxyvitamin D, Mycobacterium Tuberculosis, Epidemiology, Pulmonary

## Abstract

**Background:**

Vitamin D deficiency or insufficiency has been conducted with increased risk for tuberculosis (TB). Despite this association, discrepancies exist among different studies in different regions. The aim of this study was to evaluate the prevalence of vitamin D deficiency and its predictors in tuberculosis among the Iranian-Azari population.

**Materials and Methods:**

A total of 60 participants were enrolled in this study, 30 of which were newly diagnosed TB patients and 30 were healthy volunteers. At least two serum samples were collected, the first sample before the start of anti-TB treatment and the second sample 3 months after the effective treatment.

**Results:**

The prevalence of vitamin D deficiency (25 patients vs. 2 healthy individuals; P<0.001) and serum levels of the vitamin D (22.66±15.17 vs. 73.03±25.6 ng/mL; P<0.001) were significantly higher in patients with TB than healthy subjects. Likewise, the prevalence of vitamin D deficiency in the extrapulmonary TB group was higher than that of the pulmonary TB, but this difference was not statistically significant (P=0 .397). Moreover, there was no significant difference between mean levels of vitamin D in patients with tuberculosis before and after treatment (P = 0.787). Linear regression analysis showed there was no significant relationship between vitamin D levels after treatment and age, gender, body site of tuberculosis, and vitamin D levels before treatment, P≥0.68.

**Conclusion:**

Vitamin D deficiency is higher in patients with tuberculosis than healthy individuals.

## INTRODUCTION

Tuberculosis (TB) is a major global health problem and has been reported to cause approximately 1.4 million deaths a year (1). In addition to its negative impacts on the quality of life, it imposes a high burden on the health system (2, 3). It has been estimated that about one-third of the world’s population is infected with latent Mycobacterium tuberculosis (4), 10% of which will develop the active disease (5). Despite the decline in mortality and morbidity of the disease in recent decades, TB remains a main global public health threat (6, 7).

Several factors are considered as risk factors for tuberculosis; however, one responsible element may be vitamin D deficiency. From the past, vitamin D and sunlight were used to treat tuberculosis. Now, there is growing proof that vitamin D increases anti-mycobacterial protection (8, 9).

Vitamin D, a regulator of macrophage function, can stimulate human anti-mycobacterial activity. Many observations have shown that vitamin D deficiency is related to a higher risk of tuberculosis infection. In the first place, tuberculosis tends to occur during the cold seasons at a time when vitamin synthesis from sunlight in the skin cells is minimized and serum vitamin D levels are lower. In the next step, untreated TB patients, especially those who live in temperate climates, have lower serum vitamin D levels than healthy individuals. In addition, the prevalence of tuberculosis is higher among people such as the elderly and uremic patients who have lower serum vitamin D levels (10, 11).

The effects of vitamin D on the immune system is due to its role in the innate immunity system (12). Moreover, CD4+ and CD8+ T cells, by producing chemokines, such as CC and CXC, have protective effects against TB infection (13). The antimicrobial activity of TLRs depends on the presence of vitamin D (14). This vitamin works by binding to its nucleus receptor in the target cell. Therefore, vitamin D deficiency and any structural and functional disorders in its receptor can lead to an impairment of host immunity against bacillus tuberculosis (15). 25-hydroxyvitamin D is a biomarker for determining the vitamin D levels of the human body (16). Studies have shown that vitamin D enhances phagocytosis of macrophages and the production of Cathelicidin antimicrobial peptide and accelerates M. tuberculosis intracellular death (17). Studies have shown that there is a relationship between the deficiency of this vitamin and TB (18, 19). Also, an antimicrobial pathway depend on INF-γ in macrophages is associated with appropriate serum levels of vitamin D (20). The vitamin D levels in patients with TB have been controversial in previous studies. Some studies have reported the reduction of vitamin D levels in patient with TB compared to the healthy individuals (21, 22), while others fail to detect these findings (23). Levels of vitamin D vary among populations and affected by various racial, cultural and geographical causes (24, 25). Hence, the aim of this study was to evaluate the level of vitamin D and its confounding factors in patients with TB and healthy individuals in Iranian-Azari population.

## MATERIALS AND METHODS

Following the approval from the local Research Committee, between February 2017 and January 2018, a cross-sectional study was performed to investigate the serum vitamin D levels in patients with TB and compare them with healthy subjects. A total of 60 participants were enrolled in the study, 30 of which were newly diagnosed TB patients and 30 were healthy volunteers. Changes in vitamin D serum level were monitored during the treatment. This study was performed at a tertiary referral and academic hospital in Tabriz, East Azerbaijan province, North West of Iran. The province extends from 38° 4′ 35.76″ N and 46° 16′ 48″ E. The climate of this province is generally cold and dry, but due to its different topography, it has different climates. The inclusion criteria for patients were newly diagnosed TB patients aged above 18 years, patients who did not take vitamin D supplements, cases with confirmed diagnosis of TB that is evaluated by positive smear, polymerase chain reaction (PCR) or culture of M. tuberculosis in sputum or other body specimens. Healthy individuals who were over 18 years old, did not have any history of TB or pathologic findings in radiology, and had not taken vitamin D supplements were used in control group. These volunteers were matched to patients by age and gender. Patients and healthy individuals with HIV, a parathyroid disorder, vitamin D supplements intake, malabsorption and current infectious or an inflammatory disease along with patients that previously undergoing TB therapy were excluded from this study. Patients were informed about their inclusion in the study and signed a consent form. Patients information associated with serum vitamin D levels, such as age, gender, history of comorbidity and history of drug usage were collected.

Serum samples were collected between 7 and 9 AM after at least 12 h night fasting. In each patient, at least two vitamin D serum samples were determined and this included a sample before the onset of anti TB treatment and another one taken 3 months after treatment onset if they respond to treatment. Considering the mentioned condition, a single blood sample was taken from the control group. The response to treatment was evaluated by clinical evaluation, bacteriological examination, and chest radiograph. Vitamin D levels were determined as a serum level of total 25-hydroxyvitamin D by electrochemiluminescence binding assay (ECLIA). The assay measurement range was 4–100 ng/mL. In this study, vitamin D deficiency was defined as vitamin D (25-OH) ≤20 ng/mL, and vitamin D insufficiency as 21–29 ng/mL (26) .

For the description of the data, mean ± standard deviation was used. The variables were analyzed using Chi-square, Cramer’s V, Pearson’s and Spearman’s correlation analysis. To evaluate the difference and compare between the two groups, independent samples t-test and paired samples test were performed, respectively. The statistical analysis was performed using SPSS ver23. P<0.05 was defined as a statistically significant.

## RESULTS

**Figure 1.**
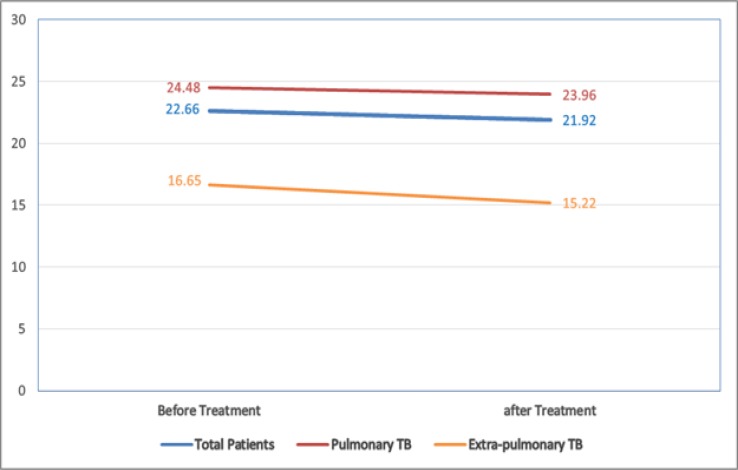
Vitamin D levels before and after treatment (ng/mL)

A total of 30 newly diagnosed patients and 30 healthy subjects were enrolled in this study. The mean age of the patients group was 38.5±11.7 and healthy subjects was 32.3±20.3 years old. Each group comprised 17 men (56%) and 13 women (43.3%). In the patient’s group, 23 patients (76.7%) had pulmonary TB and 7 patients (23.3%) had extrapulmonary TB. The serum levels of vitamin D in both groups are shown in Table 1. The mean serum vitamin D levels in the control subjects were higher than TB patients (73.03±25.6 vs. 22.66±15.17 ng/mL). Independent Samples T-test showed that this difference was statistically significant (P<0.001). The prevalence of vitamin D deficiency in the extrapulmonary TB group was higher than that of the pulmonary TB. However, Chi-square statistical analysis of this difference was not statistically significant (P=0.397). As shown in Figure 1, the mean serum level of vitamin D before treatment in patients with pulmonary TB was higher than those with extrapulmonary TB, though it was not statistically significant (P=0.238). The results of the Paired Samples Test showed that there was no significant difference between the mean serum vitamin D level in patients with TB before and after treatment (P = 0.787). The mean serum vitamin D level in patients with TB after treatment was significantly lower than that of the control subjects. Independent Samples T-test showed that this difference was statistically significant (P<0.001).

**Table 1.** Patients and control groups based on serum vitamin D level.

**Vitamin D status**	**Pulmonary TB**	**Extra Pulmonary TB**	**Control group**	**All**
Deficiency	3(13%)	1(14.3%)	0	4(6.7%)
Insufficiency	15(64.2%)	6(85.7%)	2(6.7%)	23(38.3%)
Sufficiency	5(21.7%)	0	25(83.3%)	30(50%)
Potential Intoxication	0	0	3(10%)	3(5%)
ALL	23	7	30	60

In the study of the confounding factors, serum vitamin D levels before treatment, age, and gender were evaluated. There was no significant relationship between age (P=0.297) and gender (P=0.182) with vitamin D level before treatment. Moreover, there was no significant association between age (P=0.67), gender (P=0.683), and site of tuberculosis (P=0.081) and levels of vitamin D after treatment. Only serum vitamin D level before treatment had a significant (P=0.019) and direct correlation (r =0.452) with serum vitamin D level after treatment. In other words, patients with higher serum vitamin D levels before treatment had also higher serum level of vitamin D after treatment. Moreover, a linear regression analysis was performed to predict the value of the vitamin D. The serum level of vitamin D after treatment (e.g., age, gender, type of TB and serum vitamin D level before treatment) was used as independent variables. The result indicated that neither of these factors can predict the level of vitamin D after treatment (P≥0.068).

## DISCUSSION

In this study, the prevalence of subnormal vitamin D levels in patients with TB was significantly higher than the control group. Moreover, 13.3% of patients were vitamin D deficient while 70% were vitamin D insufficient. This hypovitaminosis is similar to the previous study conducted in China with a total prevalence of 83.1% among active TB patients (27) and in the most recent study of Brazilians among prisoners with active TB with a prevalence of 75% (28). But the percentage of patients with severe vitamin D deficiency in Chinese study is more than the findings of the present study. The present study showed that none of the control group had vitamin D deficiency and only 6.7% had insufficient vitamin D. On the contrary, higher prevalence of vitamin D deficiency in non-TB subjects (38.7 and 42%) was reported in several studies in Ethiopia (29, 30). Nevertheless, Koo et al. (23) and Ho-Pham et al. (31) showed that vitamin D deficiency was not significantly different in the patients and control group.

Another interesting result of the current study was the lower level of vitamin D in patients with extrapulmonary TB, so that all of them had subnormal vitamin D levels. Although there are several studies carried out on vitamin D levels in the patient with pulmonary TB, only a few studies have been conducted on the level of vitamin D in extrapulmonary TB patients. One of the previous well-designed studies on patients with extrapulmonary TB was performed by Pareek et al. (32). They showed that the patients with extrapulmonary TB had lower mean vitamin D (25-OH D) concentration as compared with pulmonary TB and doubling in serum vitamin D concentration significantly reduced the risk of extrapulmonary TB (32).

The results of the present study showed that the mean serum vitamin D levels in patients with TB before treatment was significantly lower than that of the controls. Also, vitamin D levels in patients reduced after treatment, but this reduction was not significant. In the other word, TB treatment did not improve the vitamin D levels. In a cohort study with a similar method of vitamin D measurement (ECLIA), the mean baseline vitamin D levels of the patients were 13.7±6.3 ng/mL and 25.7±12.7 ng/mL in the controls (33). This study showed that TB is associated with a reduced vitamin D levels. However, there are different findings on the level of vitamin D after treatment. In the present study, the findings on reduced levels of vitamin D after treatment are in line with a previous study in the Korean population (23), but in contrast with the results of Hong et al. (34) study in that population. These contrary findings among different studies might be due to the difference in dietary habits, that is, marine fish consumption (27), comorbidities in the study population (35), exposure to sunlight (36), effect of seasons (36), ethnic (37), skin color, difference in laboratory assay methods and difference in vitamin D deficiency range.

In this study, no significant relationship was found between age and gender with vitamin D levels. In contrast, several studies from Pakistan (38) and Ethiopia (30) showed a higher level of vitamin D deficiency in the female gender. This might be ascribed to pregnancy and inadequate sunlight exposure. Some studies performed on an African population showed that aging was significantly associated with vitamin D deficiency (30, 39).

This cross-sectional study had some limitations. The sample size was not large enough; however, all the newly diagnosed patients were studied in one year. Furthermore, dietary intake, clothing coverage, the seasons’ effects, and residences in the urban or rural were not considered in our study. However, our study was the first report from the North West of Iran. Therefore, it is suggested that a similar study with a larger sample size should be conducted to verify the present results, considering the limitations of this study.

## CONCLUSION

The prevalence of vitamin D deficiency in patients was significantly higher than the control group. The mean of serum vitamin D levels in patients with TB before treatment was significantly lower than that of the controls, and vitamin D levels in tuberculosis patients reduced after treatment. In this study, no significant relationship was found between age and gender with vitamin D levels.
